# Assessing the impact of gait speed on gait stability using multi-scale entropy fused with plantar pressure signals

**DOI:** 10.3389/fbioe.2024.1328996

**Published:** 2024-02-28

**Authors:** Zilei Hu, Miaomiao Li, Jiale Wei, Jing Zhao, Xiaojing Tang, Haicheng Wei

**Affiliations:** ^1^ School of Electrical and Information Engineering, North Minzu University, Yinchuan, China; ^2^ School of Information Engineering, Ningxia University, Yinchuan, China; ^3^ School of Science, Ningxia Medical University, Yinchuan, Ningxia, China

**Keywords:** regional fusion pressure, multi-scale entropy, walking speed, gait stability, distribution of the plantar pressure

## Abstract

**Introduction:** Walking speed can affect gait stability and increase the risk of falling.

**Methods:** In this study, we design a device to measure the distribution of the plantar pressure to investigate the impact of the walking speed on the stability of the human gait and movements of the body. We fused the entropy acquired at multiple scales with signals of the plantar pressure to evaluate the effects of the walking speed on the stability of the human gait. We simultaneously collected data on the motion-induced pressure from eight plantar regions to obtain the fused regional pressure. To verify their accuracy, we obtained data on the plantar pressure during walking by using the force table of the Qualisys system. We then extracted the peak points and intervals of the human stride from pressure signals fused over three regions, and analyzed the mechanics of their regional fusion by using the regional amplitude–pressure ratio to obtain the distribution of the plantar pressure at an asynchronous walking speed. Furthermore, we introduced multi-scale entropy to quantify the complexity of the gait and evaluate its stability at different walking speeds.

**Results:** The results of experiments showed that increasing the speed from 2 to 6 km/h decreased the stability of the gait, with a 26.7% increase in the amplitude of pressure in the region of the forefoot. The hindfoot and forefoot regions were subjected to the minimal pressure at a speed of 2 km/h, while the most consistent stress was observed in regions of the forefoot, midfoot, and hindfoot. Moreover, the curve of entropy at a speed of 2 km/h exhibited a slow decline at a small scale and high stability at a large scale.

**Discussion:** The multi-scale entropy increased the variation in the stability of the synchronous velocity of walking compared with the sample entropy and the analysis of regional fusion mechanics. Multi-scale entropy can thus be used to qualitatively assess the relationship between the speed and stability of the gait, and to identify the most stable gait speed that can ensure gait stability and posture control.

## 1 Introduction

Walking exercise is beneficial to health, and appropriate exercise intensity can reduce the risk of chronic complications (Piercy et al., 2018; Liao et al., 2019). A rapid gait may affect a person’s stability while they are walking (McAndrew Young and Dingwell, 2012), and can lead to falls among elderly people (Nascimento et al., 2022). It can also damage the plantar soft tissue to cause foot ulcers (Wu et al., 2020). Therefore, it is important to investigate the impact of the walking speed on the plantar pressure and people’s gait to avoid injuries among elderly people.

Deep learning algorithms are being used in prevalent research to investigate the link between the plantar pressure and the human gait. A complete gait is generated as one walks with one heel on the ground until the same heel comes into contact with the ground again (Okawara et al., 2022; Caldas et al., 2017). Jeong et al. (2017) used the multi-class support vector machine to identify the plantar pressure of people walking on level ground as well as up and down a flight of stairs, and were able to classify their gait with an accuracy of 95.2%. Luo et al. (2019) measured electromyography signals of the thigh muscle and signals of the plantar pressure, and used a combination of the Long Short Term Memory (LSTM) network and the Multi-Layer Perceptron (MLP) to identify the phases of their gait with an accuracy of 94.10%. Jun et al. (2021) input sequential 3D data on the human skeleton and data on the average plantar pressure into the coding layers of the RNN and CNN, respectively, extracted the relevant features from them, and fed them into the fully connected layer of the network for classification. The two networks were able to identify abnormal gait with accuracies of 68.82% and 93.40%, respectively. Shalin et al. (2021) used data on the plantar pressure of patients with Parkinson’s disease as they walked, extracted the relevant features, and used the LSTM to detect the Parkinsonian freezing of gait with an accuracy of 95%.

Previous studies in the area have identified distinct types of gait based on clinical diagnoses, but little research has addressed the effects of the speed of the gait on its stability. Studies have shown that the complex stability of the human gait can be investigated by analyzing the time series of the interval of strides (Prakash et al., 2018). Warlop et al. (2016) found that the variation in the duration of strides affects the stability of gait in patients with Parkinson’s disease. Chandrasekaran et al. (2022) used the Lyapunov exponent to analyze the stride intervals, found that it was correlated with variations in the duration of strides, and used this to obtain the threshold of gait stability. Aziz and Arif (2006) claimed that the stride interval of the gait reflects a law of the human gait, and analyzed the complex stability of gait in patients with neurodegenerative diseases based on the symbolic entropy of the stride interval. Yu et al. (2017) proposed that the analysis of the symbolic entropy of the time series of stride intervals can reflect the complex stability of the gait. However, the above studies have used single-scale sign entropy to analyze the complex stability of the gait, where this cannot explain differences in the complex stability of the gait at the multiple time scales that are inherent in the corresponding time series.

In this study, we design a device to acquire the distributed plantar pressure to examine the effects of the speed of walking on the stability of the human gait. We propose a method for the mechanical analysis of the complex stability of the human gait based on regional fusion to this end. This device can simultaneously measure the distribution of the dynamic pressure at eight plantar locations, partition the human gait cycle, and extract the characteristics of the stride intervals by using fused values of the plantar pressure. It represents the heel-to-heel movement, full foot on the floor, the stance of the forefoot, and the toe-off in the support phase as the peaks and valleys of the waves. Following this, we introduce the ratio of the regional amplitude of the fused pressure to examine the difference in the distributions of the plantar pressure under an asynchronous speed of walking. Multi-scale entropy is used to analyze the stride interval, explain the difference in entropy at different speeds of walking at multiple time scales, quantify the complex stability of the human gait, and distinguish between its states of stability. This method can be used to evaluate the stability of the gait and the distribution of the plantar pressure at different walking speeds, and can provide a theoretical basis for determining an appropriate walking speed for rehabilitation exercises.

### 1.1 Overall structure

**FIGURE 1:**
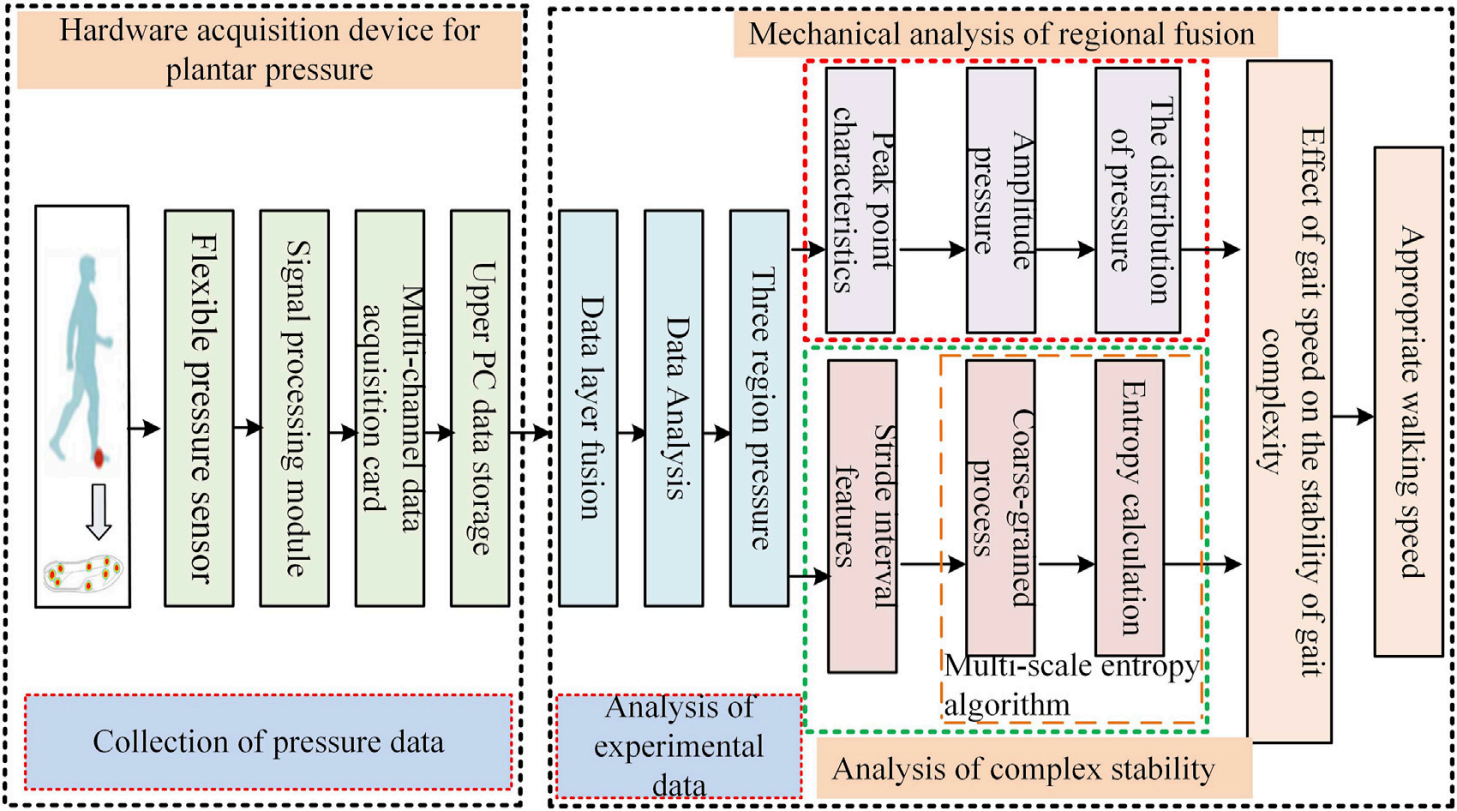
Block diagram of the overall framework to assess gait stability.

The framework design to assess the stability of the human gait based on the distribution of the plantar pressure is shown in Figure 1. Data on the dynamic plantar pressure of healthy people at different walking speeds were first collected by using a hardware acquisition device. The pressure signals from eight plantar regions were then fused to obtain the pressure distributions of regions of the hindfoot, midfoot, and forefoot. The plantar pressure was analyzed by using regional fusion mechanics and complex stability. The distributions of the plantar pressure and multi-scale entropy were estimated to assess the stability of the gait at different walking speeds, and the appropriate walking speed was then chosen to improve gait stability.

## 2 Methods

### 2.1 Hardware acquisition device for plantar pressure

**FIGURE 2:**
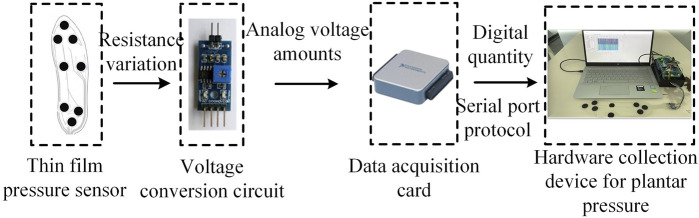
Structural composition of device used to acquire the plantar pressure.

Acquiring data on the plantar pressure required choosing an appropriate pressure sensor. Obtaining reliable data required that the normal movement of the human body not be impeded during the measurements. We designed a pressure insole with eight area pressure sensors for the measurements. The flexible thin-film resistive pressure sensor used here was based on the FSR-402 sensor. Its resistance decreased when a large force was applied to the sensing surface. The sensor had an average service life of over one million presses, a thickness of 0.46 mm, a working voltage of 5 V, and a range of accurate weight measurements of 100 to 10 kg. It converted the pressure signals on the applied surface into changes in the electrical resistance to detect the plantar pressure as a person walked. The circuit for voltage conversion transformed the resistance of the sensor into a change in the analog voltage. Data conversion was carried out by using the NI-6001 multi-channel data acquisition card. Its built-in 14-bit ADC, with a rate of sampling of up to 20 kS/s, could provide eight channels each for the analog input and the signal output. After connecting the acquisition card to an upper PC, we set-up the serial communication protocol and the DAQ driver, adjusted the frequency of sampling to 100 Hz, and stored and displayed signals of the plantar pressure as shown in Figure 2.

### 2.2 Collection of experimental data

We recruited 32 healthy male subjects, with an average foot size of 40 ± 0.74, average weight of 57.5 ± 2.55 kg, average height of 173 ± 2.45 cm, and average age of 23.5 ± 0.85 years for our experiments. None of the subjects suffered from any walking dysfunction or foot deformity. They were asked to strap the hardware device to acquire plantar pressure signals to their right calves, and wore flat shoes with sensor insoles.

The subjects were asked to walk on a treadmill at speeds of 2, 4, and 6 km/h, respectively. Before each set of measurements, we asked the subjects to walk for 1 min on the treadmill to allow them to become accustomed to its speed, and this was followed by the collection of pressure-related data at various speeds for 3 min. This experimental process was repeated several times to obtain multiple groups of experimental data. We obtained about 160,000 data points on the subjects in each group for experimental analysis, for a total of about 6,040 complete gait cycles.

**FIGURE 3:**
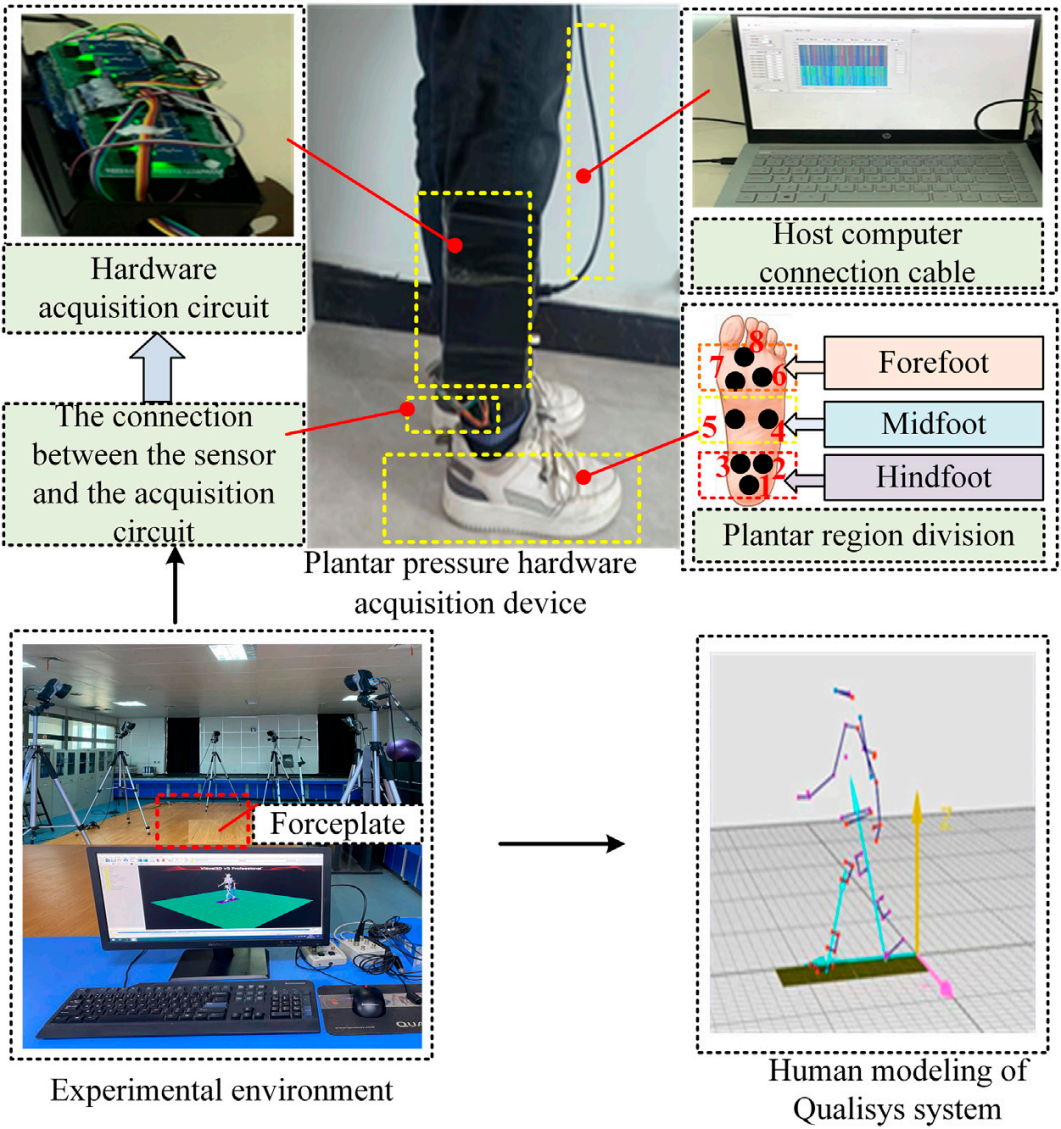
Collection of experimental data.

To validate the accuracy of the data thus obtained, we used the 3D optical motion capture system Qualisys with eight infrared cameras and two Kistler force gauges to collect data on the plantar pressure as the subjects walked on the treadmill. A force table was embedded into the ground in a longitudinal arrangement. A metronome was used during the experiment to guide the subjects to walk at the specified speed. The first step of the standing subject landed on the first force board, followed by the second step landing on the second force board. Figure 3 shows the collection of the experimental data.

### 2.3 Multi-scale entropy algorithm

The entropy is used in signal analysis to describe the complexity of the signal and represent the degree of chaos in the system. Sample entropy reflects the complexity of the system on a single scale, and cannot be used to fully quantify its complexity. The multi-scale entropy method was proposed by Costa et al. (2002), and offers the advantages of the sample entropy while avoiding the loss of information caused by the use of a single scale. We used pressure-related data from the sensor for the hindfoot region based on the data on plantar pressure, and then applied mean processing to obtain pressure signals for it. The stride interval used here was based on the interval between adjacent peak points of the pressure signals in the region of the hindfoot. The stride interval was used according to the original time series of the model of multi-scale entropy. Multi-scale entropy (MSE) can be divided into the coarse-graining of the signals and the calculation of a new sequence of entropy values (Busa and van Emmerik, 2016).

Coarse graining process:

(1) The time series of the N original signals $\text{X}=\left[\chi_{1},\chi_{2},\chi_{3},...,\chi_{\text{N}}\right]$ is coarse-grained to construct a new time series.
(2) When the scale is s=1 , the coarse-grained series is the original time series. When s=2 , let the window of length two move forward on the original sequence. Calculate the average of $\chi_{1}$ and $\chi_{2}$ to obtain $y_{1}$ .
(3) Move the window forward by two units. $y_{2}$ is obtained by calculating the average of $\chi_{3}$ and $\chi_{4}$ . Shift the window by two units once again to obtain the average value and use it to form a new sequence. The new sequence at scale s=2 is Y=y1,y2,y3,...yN2 .
(4) Similarly, when the scale s=3 , let the window of length three move forward on the time series X of the original signals. Start by averaging $\chi_{1}$ , $\chi_{2}$ and $\chi_{3}$ to $y_{1}$ . Move the window by three units, and calculate the mean value of the original sequence in the window to obtain $y_{1}$ , $y_{2}$ , and $y_{3}$ . The new sequence at scale s=3 is then Y=y1,y2,y3,...yN3

Calculating the entropy of the new sequence:

(1) Suppose that the length of the time series X of the original signals is N and the scale factor is *s*. Then, the coarse-grained sequence is given by:

$$Z_{j}^{\left(s\right)}=\frac{1}{s}\sum_{i=\left(j-1\right)s+1}^{js}x\left(i\right),1\leq j\leq \frac{N}{S}$$ (1)

(2) Under an *m*-dimensional vector, the data sequence is given by:

$$Z\left(i\right)=\left[z_{i},z_{i+1},z_{i+2},...z_{i+m-1}\right],1\leq i\leq \frac{N}{s}-m+1$$ (2)

(3) Find the number of distances $d_{ij}$ shorter than *r*, $d_{ij}<r$ . Then, the ratio of the number of such distances to the total number of distances is given by:

$$d_{ij}\left[Z\left(i\right),Z\left(j\right)\right]=\max_{0<k<m-1}\left[z\left(i+k\right)-z\left(j+k\right)\right]$$ (3)

$$L_{i}^{m}\left(r\right)=num\left(d_{ij}<r\right)/\left(N/s-m\right),i\neq j,i=1,...,\frac{N}{s}-m+1$$ (4)

where $d_{ij}$ is the maximum distance between vectors $Z\left(i\right)$ and $Z\left(j\right)$ , and *r* is the range of tolerance for a given time series.

(4) Set the number of dimensions to *m* + 1 and repeat the above steps to obtain the following:

$$L_{i}^{m+1}\left(r\right)=num\left(d_{ij}<r\right)/\left(N/s-m\right),i\neq j,i=1,...\frac{N}{s}-m$$ (5)

(5) Calculate the average of $L_{i}^{m}\left(r\right)$ and $L_{i}^{m+1}\left(r\right)$ :

$$L_{i}^{m}\left(r\right)=\frac{1}{N/s-m+1}\sum_{i=1}^{N/s-m+1}L_{i}^{m}\left(r\right)$$ (6)

$$L_{i}^{m+1}\left(r\right)=\frac{1}{N/s-m+1}\sum_{i=1}^{N/s-m}L_{i}^{m+1}\left(r\right)$$ (7)

(6) The entropy value of the new sequence is that of the MSE, $E_{MSE}$ :

$$E_{MSE}=-\ln\left(L^{m+1}\left(r\right)-L^{m}\left(r\right)\right)$$ (8)

Some studies have shown that too large a number of dimensions *m* significantly increases the amount of required computation and leads to a decline in computational efficiency. *m* is generally set to one or two, while *r* is set to 0.10–0.25SD, where SD is the standard deviation of the original time series. When *m* is two, the length of the sequence N is minimally dependent on the accuracy of the calculated results (Zheng et al., 2023). Therefore, we set m=2 and r=0.25SD in this study.

## 3 Results

### 3.1 Validation of experimental data

A gait cycle is divided into a stance phase and a swinging phase. The stance phase is the process in which the foot makes contact with the ground to generate plantar pressure. The swing phase is defined as the forward movement of the limb without any contact with the ground (Cicirelli et al., 2022).

**FIGURE 4:**
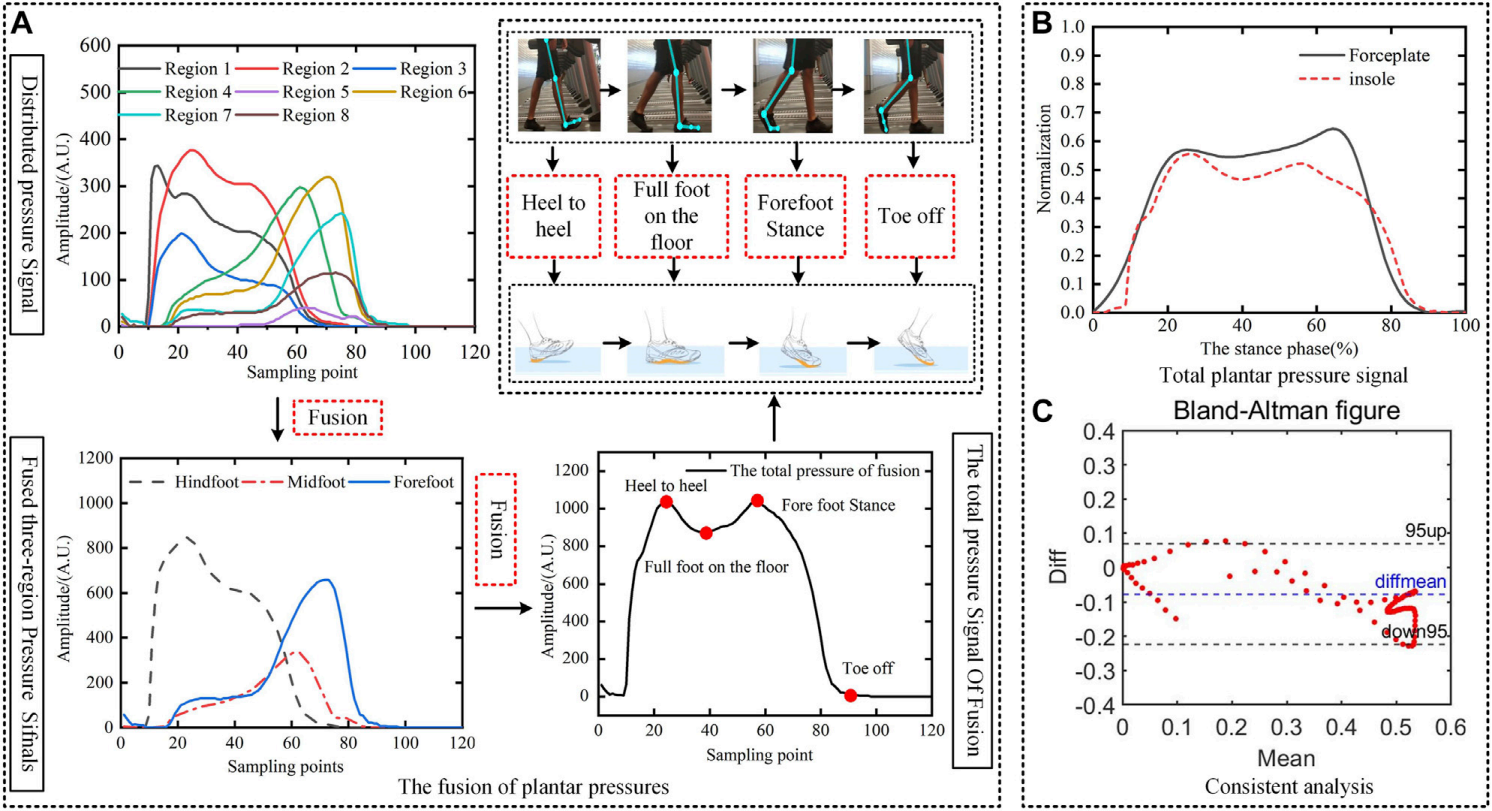
Comparative analysis of signals of plantar pressure measured at the pressure plate and the insole. **(A)** The fusion of plantar pressures. **(B)** Total plantar pressure signal. **(C)** Consistent analysis.

A comparative analysis of signals of the plantar pressure measured by the pressure plate and the insole is shown in Figure 4. We use a speed of 2 km/h as an example. The pressure signals at eight points in the plantar as the subject walked were obtained and fused. Figure 4A shows that the force at each point exhibited a peak of the wave as the foot came into contact with the ground. The pressure-related data from each sensor in the three regions were averaged and fused. The fused pressure signals showed the changes in pressure in the hindfoot, midfoot, and forefoot throughout the stance phase. The total plantar pressure was obtained by further fusing these three regional signals, and can be used to illustrate the troughs and peaks of the four states in the stance phase.

To verify the accuracy of the pressure-related data obtained from the insole, we eliminated the influence of the subject’s weight on the distribution of the plantar pressure. The fused signals of the total pressure obtained from the insole and the pressure plate of the same subject were normalized based on their amplitude and the transverse axis, respectively. Figure 4B shows that the trends of changes in the pressure signals of both were consistent with each other. The reaction force from the vertical ground obtained by the pressure plate and the insole had a prominent “double peak” characteristic.

We used the Bland–Altman plot to evaluate the consistency of the two methods of measurement. Figure 4C shows that the difference between the measurements of the pressure plate and the insole was within the 95% confidence interval. *p* < 0.05 and r = 0.9513 for these two methods of measurement. These results show that there was a significant correlation between the data measured from the insole and the pressure plate, which leads us to conclude that they were reliable. The second peak point of measurements of the insole was smaller than that of the force measurement table. This is because when the sole was in the forefoot stance, it made full contact with the ground and there were few pressure sensors in the sole area of the insole. As a result, the pressure distribution in the sole of the foot could not be entirely monitored, and a smaller amount of pressure-related data were obtained from it.

### 3.2 Mechanical analysis of regional fusion

To eliminate the influence of the subject’s weight on the experimental results, we normalized the amplitudes of pressure of the three plantar regions, which were fused in the stance phase of the gait cycle, by weight. We used the ratio of the amplitude of pressure to the weight of the subjects to examine the differences in distributions of the plantar pressure at walking speeds of 2, 4, and 6 km/h.

**FIGURE 5:**
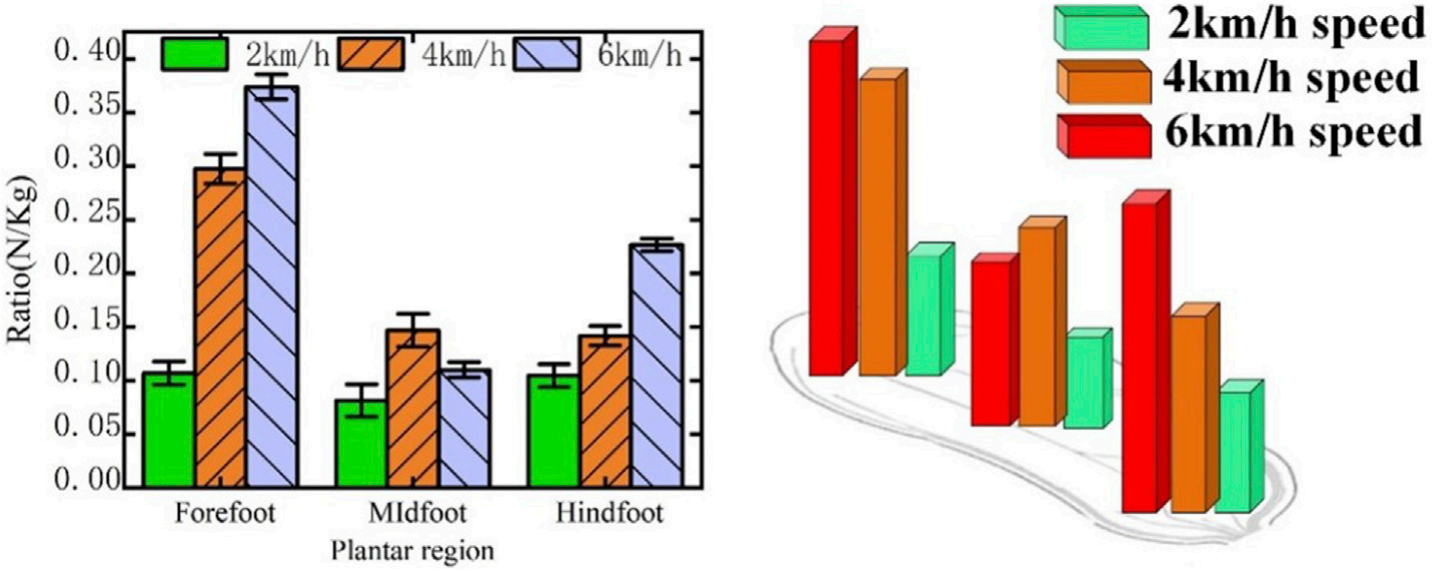
Changes in pressure in the three plantar regions at three walking speeds.

Figure 5 shows the pressures in the three plantar regions at the three speeds of walking considered here. As the walking speed increased, the ratios of the amplitude of pressure in the hindfoot and forefoot regions of the body increased significantly. When the subject’s walking speed was increased from 2 to 6 km/h, the ratio of the amplitude of pressure in the forefoot region increased by 26.7%. The foot bears the weight of the body during normal walking, while balance and movement are controlled through contractions of the plantar muscles. If the plantar pressure is not regularly distributed, the body requires more control to maintain balance and stability during normal walking.

Studies have shown that increased plantar pressure can enhance the risks of soft tissue injury in the plantar and metatarsal stress fracture (Zhang et al., 2018). The forefoot area is responsible for the balance and control of the center of gravity of the body. Nevertheless, the pressure on the forefoot is excessively high such that the body requires greater control to maintain equilibrium (Zheng et al., 2020). When the subjects walked at a speed of 2 km/h, the pressure in the forefoot and hindfoot regions was relatively low, and its distribution in the three regions was the most uniform. When they walked at 4 km/h, the magnitude of force in the forefoot and hindfoot areas progressively grew, the load on the hindfoot and forefoot gradually increased, and the disparity in the pressure distributions at the three locations became larger. When the subjects walked at 6 km/h, the magnitude of force in the forefoot and hindfoot regions continued to increase, the pressure distributions in the three regions were the largest, and might have led to an increase in gait oscillation to lead to an unstable gait. Thus, the pressure distribution in the three plantar zones was reasonably balanced at a speed of 2 km/h, and the burden on the hindfoot and the forefoot was the smallest. This helped the balance of the body and the health of the feet.

### 3.3 Analysis of complex stability

The differences in the pressure distributions in the three plantar regions caused by an asynchronous speed of walking cannot directly reflect the corresponding differences in the stability of the gait. Studies have shown that the irregularity of distribution of the plantar pressure can be reflected by the varying degrees of changes in the length and frequency of the stride, which in turn affect the stability of the gait (Biswas et al., 2008). We used MSE to examine the time series of stride intervals of the subjects to measure the complexity of the rhythm of their gait at different time scales, and thus to evaluate the differences in its stability at different speeds of walking.

A system with a larger entropy is more complex and less regular (Kędziorek and Blażkiewicz, 2020). Table 1 shows that walking at 2 km/h yielded the lowest overall sample entropy of the gait, indicating that the sequence of strides had the highest self-similarity, the least complexity, and led to a highly regular gait in this case. However, the differences among the three were not prominent, and the overall difference in entropy was small.

**TABLE 1:** Values of entropy of gaits at three speeds.

Index of complexity	2 km/h	4 km/h	6 km/h
SampEn	0.816 ± 0.04	0.907 ± 0.07	1.088 ± 0.09
MSE_S_	0.901 ± 0.03	1.216 ± 0.02	1.480 ± 0.04
MSE_L_	0.732 ± 0.02	0.773 ± 0.03	0.89774 ± 0.02

Note: The values are expressed as mean ± SD. “SampEn” is the overall sample entropy of the gait. “MSE_S_” is the mean value of scales 1–3. “MSE_L_” is the mean value of scales 4–6.

**FIGURE 6:**
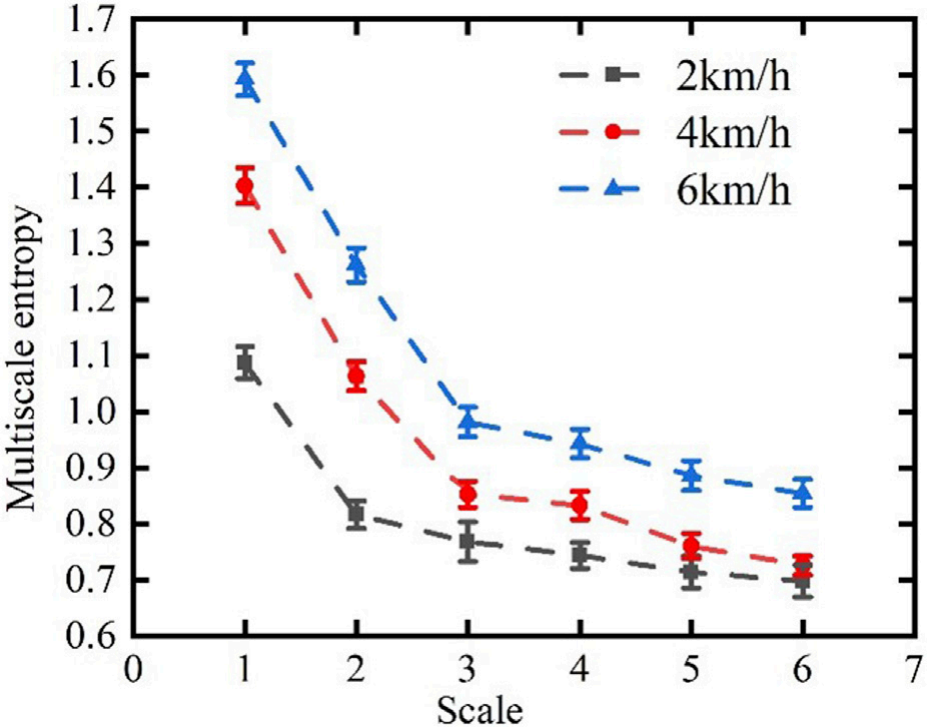
Curves of distribution of the multi-scale entropy of the stride interval of the gait.

Figure 6 shows curves of the distribution of MSE as the subjects walked at the three speeds considered here, while Table 1 shows the scale of the magnitude of entropy of the gait. MSE can be used to amplify the temporal differences in gait complexity at the three speeds. Compared with those at 4 km/h and 6 km/h, the MSE at a speed of walking of 2 km/h was smaller by 0.315 and 0.579, respectively, and this shows that the differences in entropy among the three speeds was prominent at a small scale but slight at a large scale. MSE was thus able to more clearly identify the differences in stability at various walking speeds, and to amplify the differences in entropy at a small scale in comparison with sample entropy. This may be because the stability and regularity of the gait are impaired over short time scales, but the change in the stride interval is generally smooth and stable over long time scales as the body gradually adapts to the change in the frequency of steps.

Therefore, the curve of entropy at a small scale decreased more slowly at 2 km/h than at 4 km/h and 6 km/h, and was the most stable at a large scale.

## 4 Discussion

Some studies have shown that the more stable the gait is, the lower are the multi-scale entropy of the sequence of stride intervals and the rate of decline (Hsieh and Abbod, 2021). When one is walking slowly, the frequency of strides is low, the stride interval is relatively long, and the gait is more stable because the difference between steps is relatively small and body control is thus easier (Wu et al., 2019). England and Granata quantified the stability of the gait by using the Lyapunov exponent λ, and found that λ was smaller at lower walking speeds. This indicates that slower walking increases the stability of the gait (England and Granata, 2007). The subjects in our experiments exhibited a more dynamically stable gait at lower speeds, and older adults at risk of falling are advised to reduce their walking speed to improve their stability (Dingwell and Marin, 2006). Walking quickly may result in a less regular gait and a more complex time scale owing to the increased frequency of steps and the shortening of the stride interval, where this reduces the stability of the gait and increases the difficulty of body control. Therefore, walking at a speed of 2 km/h is more conducive to the postural balance and health of the body than walking at speeds of 4 and 6 km/h.

## 5 Conclusion

The results of this study showed that different walking speeds have significant effects on the distribution of the plantar pressure and the stability of the human gait. Through an analysis of the human gait based on the plantar pressure, we combined the mechanical analysis of regionally fused data with complex stability analysis based on multi-scale entropy to differentiate between the stabilities of the gait at different speeds of walking at multiple time scales. The results of experiments involving subjects walking at speeds of 2, 4, and 6 km/h showed that the differences between gait stability were prominent at small scales but weak at large scales. This shows that the stability of the gait may be compromised at short time scales. A walking speed of 2 km/h yielded a lower complexity than the other two speeds considered here, and the curve of entropy decreased more slowly at a small scale. This curve was the most stable at a large scale, and this reflected a stable gait. By distinguishing between the stabilities of the gait at asynchronous speeds of walking, the proposed method can help clinicians develop training programs to help patients balance their gait and reduce the risk of falls among the elderly.

## Data Availability

The original contributions presented in the study are included in the article/Supplementary Material, further inquiries can be directed to the corresponding author.
